# Supplementary material for: Dynamic pricing optimization for high-speed railway based on passenger flow assignment
Source: PLoS One. 2024 Dec 17;19(12):e0314713. doi: 10.1371/journal.pone.0314713 (PMC11651575; doi:10.1371/journal.pone.0314713)
Supplement: Attachment — Submitted filename: Response to Reviewers.docx [file pone.0314713.s003.docx]

Dear editor and reviewers,

On behalf of my co-authors, we thank you very much for giving us an opportunity to revise our manuscript. We appreciate editor and reviewers very much for their positive and constructive comments and suggestions on our manuscript entitled "Dynamic Pricing Optimization for High-Speed Railway Based on Passenger Flow Assignment" (PONE-D-24-26027).

These comments are very valuable and helpful for the improvement of our paper, and also have important guiding significance for our research. We have studied comments carefully and have made correction which we hope meet with approval.

We are very sorry to update the revised manuscript so late because of the case study redesign, calculation and analysis.

Note: In the response letter, the explanatory text is in blue, the revised content in the manuscript is in green. ‘L’ shows the index of lines in the ‘Revised Manuscript with Track Changes’.

Best wishes,

CAO Jiren

Reviewer #1：

This paper combines the revenue management concept with the differentiated products, and puts forward a ticket price adjustment idea for dynamic pricing of high-speed railway. Through the construction of the space-time service network, a multi-objective dynamic pricing model and a hybrid solution algorithm based on passenger flow assignment are proposed. The case study proves the feasibility of the model and algorithm.

Some suggestions:

1. To sort out existing literature research using tables.

Response: Thanks for your valuable suggestion.

After adding some literatures, we have sorted out the existing literatures with a table, while modifying the innovative expression.

Table 1 Relevant literature research content statistics

| References | Revenue management | Dynamic pricing strategy | Passenger choice behavior | Passenger flow assignment | Transport product classification |
| --- | --- | --- | --- | --- | --- |
| Van Vuuren(2002); Yao, Yang and Zhang(2013); Xiaoqiang, Lang and Jin(2017) | Y | Y | N | N | N |
| Cao, Li and Vaze(2019); Li, Li and Ma(2021) | Y | N | N | N | N |
| Frejinger and Bierlaire(2007); Kusakabe, Iryo and Asakura(2010)；Zou and Nie(2014); Li, Tian and Li(2016); Li, Yao and Yamamoto(2020);Yan, Jian and Li(2022); Zhang, Zhao and Wang(2022) | N | N | Y | N | N |
| Fu, Sperry and Nie(2013); Sun, Lu and Jin(2015);Binder, Maknoon and Bierlaire(2017); Zhai, Zhao and Chen(2018); Zhang, Meng and Wang(2019); Zhang, Yao and Zheng(2020); Huan, Yao and Zhang(2021); Huang, Liang and Li(2022); Hao, Bingfeng and Zhao(2022); Yu, Liu and Fang(2023); | N | N | Y | Y | N |
| Hetrakul and Cirillo(2014); Cirillo, Bastin and Hetrakul(2018); Yan, Li and Han(2019); Hu, Shi and Xu(2020); Deng, Xu and Zeng(2021); Xu, Zhong and Hu(2022) | Y | N | Y | N | N |
| Zheng(2020); Su, Peng and Deng(2021) | Y | N | Y | Y | N |
| Jing, Guo and Chen(2022) | Y | N | Y | N | Y |
| This paper | Y | Y | Y | Y | Y |

Whether research content is included: Consider (Y); Not considered (N).

According to the requirements of the journal, all references are cited in brackets.

(Line 207-210)

The existing literatures have been comprehensively studied, including single-field and cross-field studies. The comparison of existing literatures is shown in Table 1.

Table 1 Relevant literature research content statistics

| References | Revenue management | Dynamic pricing strategy | Passenger choice behavior | Passenger flow assignment | Transport product classification |
| --- | --- | --- | --- | --- | --- |
| [4-6] | Y | Y | N | N | N |
| [7, 8] | Y | N | N | N | N |
| [9, 10, 12, 14, 19, 22, 23] | N | N | Y | N | N |
| [11, 13, 15-18, 20, 21, 24, 25] | N | N | Y | Y | N |
| [26-29, 32, 33] | Y | N | Y | N | N |
| [30, 31] | Y | N | Y | Y | N |
| [34] | Y | N | Y | N | Y |
| This paper | Y | Y | Y | Y | Y |

Whether research content is included: Consider (Y); Not considered (N).

(Line 223-232)

The innovations of this study are as follows:

1) Taking the game competition relationship and revenue management of differentiated train products into consideration, dynamic pricing is combined with passenger flow assignment. The train classification is combined linked with specific ticket price strategy, and different price adjustment ranges are set for different classes of trains.

2) The path size logit assignment based on capacity constraint-passenger flow increment accurate algorithm is nested into the large-scale neighborhood search algorithm. The exact matching of passenger flow and train flow is ensured while the heuristic iteration of ticket price is carried out. Real high-speed railway operational data are entered in case study, including passenger flow data and train timetable data.

2. Labor ratio should be less than 0.7.

Response: Thanks for your critical and valuable comments.

Based on your suggestion, we try to estimate the values of labor ratio from official data. According to "Statistical Bulletin of the National Economic and Social Development of the People's Republic of China for 2019" and "Statistical Bulletin of the National Economic and Social Development of Shandong Province for 2019", population aged 15-64 is considered as the working population, and the values of labor ratio in Shandong province and China in 2019 were 0.66 and 0.64, respectively.

More details of passenger time value calculation can be found in the response to No.8 comment of Reviewer#2.

(Line 338-353)

Since the *Shandong circular high-speed railway* not only includes the passenger flow in Shandong province, in order to ensure the availability of data, the passenger time value of China and Shandong province were calculated respectively. Table 3 is the calculation of passenger time value.

Table 3. Calculation of passenger time value

| Region | *GDP*(*trillion yuan*) | *P*(*million*) | *LT* | *LR*(*h*) | $\kappa_{w}$(*yuan/h*) |
| --- | --- | --- | --- | --- | --- |
| Shandong province | 7.05 | 10070 | 0.66 | 2190 | 48.44 |
| China | 98.6515 | 141000 | 0.64 | 2190 | 49.92 |

Data source: National Bureau of Statistics for 2019, Shandong Statistical Yearbook for 2019, Statistical Bulletin of the National Economic and Social Development of the People's Republic of China for 2019, Statistical Bulletin of the National Economic and Social Development of Shandong Province for 2019.

Table 4 is the Passenger flow of Jinan Railway Bureau in 2019. The local passengers and export passengers correspond to the passenger time value of Shandong province. The import passengers and carrying passengers correspond to the passenger time value of China.

Table 4. Passenger flow of Jinan Railway Bureau in 2019

| Passenger flow type | Number (*million*) |
| --- | --- |
| Local | 9192 |
| Export | 6011 |
| Import | 6050 |
| Carrying | 9241 |

Data source: Compilation of National Railway Statistical Data in 2019.

According to the proportion of different types of passenger flow, the passenger time value was 49.18 yuan/h, which was rounded up to 50 yuan/h.

3. To give the dimension of M (=15).

Response: Thanks for your valuable question.

*M* represents the maximum fatigue recovery time(unit h). $\tau$is a dimensionless parameter, used to describe the minimum fatigue recovery time $\frac{M}{1+\tau}$. $\delta$is the strength coefficient of fatigue recovery time (unit is h^-1^). The larger the value of $\delta$, the longer the fatigue recovery time.

For high-speed railway trains, the values of *M*, $\tau$and $\delta$ in Shi, Deng and Li(2004) were defined as 15, 59 and 0.28 respectively, published in Journal of the China Railway Society (the top journal of Chinese railway research). The above parameters values have been recognized by the Chinese transportation academic circle. In terms of journal articles, they were cited in Jing, Sun and Zhang(2020) and Zhou, Cai and Liu(2021). In terms of the dissertation, they were cited in Qiu(2019), Tang(2021), Liu(2024).

The relevant references are as follows:

[1].	Shi, F., et al., Research on Passenger Train Plans for Dedicated Passenger Traffic Lines. Journal of the China Railway Society, 2004(02): p. 16-20.

[2].	Jing, Y., J. Sun and Z. Zhang, Research on Time Differential Pricing Strategy of Intercity Railway Based on Generalized Cost Function. Journal of the China Railway Society, 2020. 42(05): p. 29-36.

[3].	Zhou, W., et al., Optimization of differentiated fare and ticket amount for high-speed train. Journal of Railway Science and Engineering, 2021. 18(04): p. 869-876.

[4].	Qiu, Y., Research on Pricing Method of High Speed Railway Passenger Ticket Based on Muti-stage Passenger Selection Characteristics. 2019, Beijing Jiaotong University.

[5].	Tang, J., Study on Operation Plan of Cross-line Trainbetween Ordinary-speed Railway and High-speedRailway Based on Passenger Choice Behavior. 2021, Southwest Jiaotong University.

[6].	Liu, Z., Research on Differentiated Pricing Strategy of High-speed Railway Trains Considering Passenger Travel Characteristics. 2024, Beijing Jiaotong University.

We have corrected the above parameters description in the article.

(Line 246)

**Table 2. Notations definition table**

| **Notation** | **Definition** |
| --- | --- |
| *M* | The maximum fatigue recovery time, *M*=15, unit is hour |
| $\tau$ | The dimensionless parameter, $\tau=59$ |
| $\delta$ | The strength coefficient of fatigue recovery time ,$\delta=0.28$, unit is h^-1^ |

(Line 354-356)

Seat comfort cost $SC_{w,k,s}$ is related to the fatigue recovery time, as shown in Eq(6). For high-speed railway trains, [35] defined the values of *M*, $\tau$and $\delta$ as 15, 59 and 0.28 respectively.

[35].Shi, F., et al., Research on Passenger Train Plans for Dedicated Passenger Traffic Lines. Journal of the China Railway Society, 2004.

4. To correct text errors: p6, "be chose"; p8," constraint, , ";p9,"、".

Response: Thanks for your valuable comments.

Text errors can detract significantly from the overall professionalism and clarity of an SCI manuscript. We sincerely apologize for our oversight in this regard.

(1) Original page 6, the grammar of passive voice has been corrected.

(2) Original page 8, the duplication of "," has been corrected.

(3) Original page 9, the punctuation problem has been corrected. We have replaced "、" with ",".

(Line 311-313)

(1) Original page 6

In the space-time service network, there are a large number of available routes for same OD at the same time, but in the actual travel process, some service routes will not be chosen.

(Line 421-423)

(2) Original page 8

Z_2_ considers multiple constraints which are related to passenger flow, including passenger choice route capacity constraint, route passenger flow non-negative constraint, OD passenger flow identically equal constraint, transfer times constraint, as shown in Eq(15)-Eq(18).

(Line 452-454)

(3) Original page 9

Step 1: Input initial data, including line price rate ***R_0_***, dynamic pricing range **[*LowerRate*, *UpperRate*]**, total iterations times ***T,*** convergence value ***△c***, OD passenger flow data, train timetable data.

Reviewer #2：This paper studied pricing problem for HSR aiming to maximize railway revenue and passenger travel benefit. The authors established a multi-objective optimization model and adopted large neighborhood search heuristic algorithm to solve it. This research is interesting. However, there are some drawbacks needing to be improved. The major comments are given below:

1. English writing needs improvement.

Response: Thanks for your valuable suggestion.

There was still room for improvement in our writing, and the use of professional words was not very accurate. After receiving the comment, we have carefully revised the language expression in the paper, and checked the full manuscript. Ambiguous phrases have been clarified, and the overall flow of the manuscript has been refined to ensure that our key messages are conveyed effectively and persuasively.

2. The existing literature about passenger choice behavior and passenger flow assignment in this study are mainly related to public transportation, urban railway transit, dockless bike sharing systems. It is suggested to review related literature in railway.

Response: Thanks for your valuable suggestion.

In response to the reviewers' suggestions, we have carefully reviewed and supplemented our manuscript with additional relevant references. In term of passenger choice behavior and passenger flow assignment, we have added five literatures related to railway.

(Line 162-163)

[10] presented an estimation method and algorithm based on long-term transaction data for railway passengers.

[10].	Kusakabe, T., T. Iryo and Y. Asakura, Estimation method for railway passengers' train choice behavior with smart card transaction data. Transportation, 2010.

(Line 164-166)

[12] analyzed the choice behavior preferences of high-speed railway passengers. The case results showed that with the increase of travel distance, passengers' sensitivity to ticket price and speed increased correspondingly.

[12].	Zou, Y.T. and L. Nie, Research on Passenger's Choice Behavior Based on Beijing-Guangzhou High-Speed Railway Ticket Data. Advances in Transportation, 2014. 505-506: p. 601-606.

(Line 168-171)

[14] used disaggregate model to study the choice behavior for traveler in comprehensive transportation corridor and found the advantage distance of high speed railway was about 500-1000 km. By adjusting ticket prices, the market shares of different mode could be effectively optimized.

[14].	Li, X., X. Tian and X. Li, Multi-mode Choice Behavior for Passenger in Comprehensive Transportation Corridor. Green Intelligent Transportation System and Safety, 2016. 137: p. 849-857.

(Line 184-188)

[23] established the conditional logit model and analyzed the ticket purchase influencing factors of business passengers and non-business passengers. Aiming at the  integrated mode of cross-line and skip-stop operation between state and suburban railway, [24] proposed a passenger flow assignment model based on the path-sized logit and designed an improved method of successive averages algorithm.

[23].	YAN, Z., et al., Modelling the Passenger Choice Behaviour of Buying High-Speed Railway Tickets. Promet, 2022. 34(3): p. 455-465.

[24].	Huang, X., et al., Research on Passenger Flow Assignment of Integrated Cross-Line and Skip-Stop Operation between State Railway and Suburban Railway. Applied Sciences, 2022. 12(7): p. 3617.

3. The summary of the existing literature cannot reflect the research gaps between them and this study. The advantages of this paper are not clear. It is suggested to add a table to compare the existing studies with this work.

Response: Thanks for your valuable suggestion.

After adding some literatures, we have sorted out the existing literatures with a table, while modifying the innovative expression.

Table 1 Relevant literature research content statistics

| References | Revenue management | Dynamic pricing strategy | Passenger choice behavior | Passenger flow assignment | Transport product classification |
| --- | --- | --- | --- | --- | --- |
| Van Vuuren(2002); Yao, Yang and Zhang(2013); Xiaoqiang, Lang and Jin(2017) | Y | Y | N | N | N |
| Cao, Li and Vaze(2019); Li, Li and Ma(2021) | Y | N | N | N | N |
| Frejinger and Bierlaire(2007); Kusakabe, Iryo and Asakura(2010)；Zou and Nie(2014); Li, Tian and Li(2016); Li, Yao and Yamamoto(2020);Yan, Jian and Li(2022); Zhang, Zhao and Wang(2022) | N | N | Y | N | N |
| Fu, Sperry and Nie(2013); Sun, Lu and Jin(2015);Binder, Maknoon and Bierlaire(2017); Zhai, Zhao and Chen(2018); Zhang, Meng and Wang(2019); Zhang, Yao and Zheng(2020); Huan, Yao and Zhang(2021); Huang, Liang and Li(2022); Hao, Bingfeng and Zhao(2022); Yu, Liu and Fang(2023); | N | N | Y | Y | N |
| Hetrakul and Cirillo(2014); Cirillo, Bastin and Hetrakul(2018); Yan, Li and Han(2019); Hu, Shi and Xu(2020); Deng, Xu and Zeng(2021); Xu, Zhong and Hu(2022) | Y | N | Y | N | N |
| Zheng(2020); Su, Peng and Deng(2021) | Y | N | Y | Y | N |
| Jing, Guo and Chen(2022) | Y | N | Y | N | Y |
| This paper | Y | Y | Y | Y | Y |

Whether research content is included: Consider (Y); Not considered (N).

According to the requirements of the journal, all references are cited in brackets.

(Line 207-210)

The existing literatures have been comprehensively studied, including single-field and cross-field studies. The comparison of existing literatures is shown in Table 1.

Table 1 Relevant literature research content statistics

| References | Revenue management | Dynamic pricing strategy | Passenger choice behavior | Passenger flow assignment | Transport product classification |
| --- | --- | --- | --- | --- | --- |
| [4-6] | Y | Y | N | N | N |
| [7, 8] | Y | N | N | N | N |
| [9, 10, 12, 14, 19, 22, 23] | N | N | Y | N | N |
| [11, 13, 15-18, 20, 21, 24, 25] | N | N | Y | Y | N |
| [26-29, 32, 33] | Y | N | Y | N | N |
| [30, 31] | Y | N | Y | Y | N |
| [34] | Y | N | Y | N | Y |
| This paper | Y | Y | Y | Y | Y |

Whether research content is included: Consider (Y); Not considered (N).

(Line 223-232)

The innovations of this study are as follows:

1) Taking the game competition relationship and revenue management of differentiated train products into consideration, dynamic pricing is combined with passenger flow assignment. The train classification is combined linked with specific ticket price strategy, and different price adjustment ranges are set for different classes of trains.

2) The path size logit assignment based on capacity constraint-passenger flow increment accurate algorithm is nested into the large-scale neighborhood search algorithm. The exact matching of passenger flow and train flow is ensured while the heuristic iteration of ticket price is carried out. Real high-speed railway operational data are entered in case study, including passenger flow data and train timetable data.

4. The ticket price of OD w to seat s in route k is the decision variable in this paper. The authors mentioned that the trains are decomposed into different classes. However, the subscript index of the decision variable does not include the train. It seems contradictory. In the numerical studies, the authors mentioned that only second-class seats are considered in case due to the availability of data. Thus, s is equal to 1 and the subscript s (seat s) does not seem to make sense.

Response: Thanks for your valuable question.

In terms of the decision variable, in the train service network based on timetable, the routes should include not only the direct routes, but also the transfer routes. The direct route contains only one train, and the transfer route contains multiple trains. With this in mind, we chose the route(route *k*) rather than the train when setting the decision variable.

The following will explain in detail how the algorithm searches for direct routes and transfer routes. Since this part was not the key innovation of the article, we did not include it in the manuscript.

(1) Search direct routes. For the current OD, all trains are traversed to search direct arcs between departure station and arrival station. If the train meets the following constraints, it will be promoted to one direct route and added to direct routes set.

1) The train serves departure station and arrival station simultaneously.

2) The order of departure station is earlier than arrival station.

(2) Search transfer routes. For the current OD, all trains are traversed to search transfer arcs between departure station and arrival station. If the trains meet the following constraints, they will be merged into one-transfer route and be added to one-transfer routes set.

1) The first train serves departure station and a transfer station.

2) The order of departure station is earlier than the transfer station for first train.

3) The second train serves the same transfer station and arrival station.

4) The order of transfer station is earlier than arrival station for second train.

5) At the transfer station, the arrival time of first train is earlier than the departure time of second train.

6) At the transfer station, the time interval meets the minimum transfer time. Depending on the actual situation, it takes 10-20 minutes for passengers to transfer at stations with convenient transfer equipment. Therefore, the minimum transfer time is conservatively set to 30 minutes.

In terms of numerical studies, we considered the influence of seats in the initial modeling, aiming to propose a model that includes OD, route and seat differentiation. What we did not expect was that the current data on Chinese railway was too difficult to obtain, and we could only get the data on second-class seats.

According to the model and algorithms proposed in this paper, different seats of the same OD and the same train correspond to different decision variables(i.e. ticket price *p*), which belongs to the superposition of workload. This means that the second-class seat scenario case can still verify the applicability of the model and algorithms. The method proposed in this paper can still be applied to the scenarios considering different seats.

In the meantime, we promise that as long as the data allows, we are committed to improving and publishing the cases involving seats in the follow-up research.

As for the confusion that may be caused by the decision variable and numerical studies, we have made supplementary explanation in the paper.

(Line 246)

Table 2. Notations definition table

| Notation | Definition | Remark |
| --- | --- | --- |
| ***OD*** | Passenger flow ODs set | $w\in OD$ |
| ***Route*** | Reasonable service routes set, containing direct routes(i.e. one train) and transfer routes(i.e. multiple trains) | $k,j\in Route$ |

(Line 401-405)

As the decision variable of dynamic pricing problem, the ticket price *p* is related to the line price rate per unit distance *p_0_* and the distance of current OD. *p* changes with the change of *p_0_*, and then changes the reasonable service route utilities *c* and passenger flow assignment results *f*. It should be noted that since the reasonable service routes contains direct routes and transfer routes, the subscript of *p* uses route(i.e. subscript *k*) instead of train.

(Line 570-576)

Part of actual train timetable data and passenger flow data on October 25 in 2019 is selected as the case input, including 12 circular high-speed railway train and covering 1050 ODs. Due to the availability of data, only second-class seats are considered in case. It should be noted that different seats of the same OD and the same train correspond to different decision variables p, which belongs to the superposition of workload. The second-class seat scenario case can still verify the applicability of the model and algorithms which can still be applied to the scenarios considering different seats.

5. The clarity of each figure in this paper needs to be enhanced.

Response: Thanks for your valuable suggestion.

According to the official website of *plos one*, the figures requirements are as follows.

The specific website of PACE is [Home Page - Apex.PACE (apexcovantage.com)](https://pacev2.apexcovantage.com/).

In the initial manuscript, all figures(TIFF format) were formatted by the above website to ensure that they met the basic requirements of *plos one*.

In the 'Revised Manuscript with Track Changes' and 'Manuscript', we have guaranteed the conformity of the figures. All figures are 400ppi resolution.

In the 'Response to Reviewers', in order to ensure the clarity of figures, we use the vector figures in VSDX format and summarize all figures as a whole below.

Fig 1. High-Speed Railway Passenger Flow Assignment Process

Fig 2. Sample of train service network

Fig 3. Abstracted train service network

Fig 4. Chart of changes in passenger satisfaction

Fig 5. The overall algorithm structure

Fig 6. Path Size Logit Assignment based on Capacity Constraint-Passenger Flow Increment Algorithm

Fig 7. The schematic diagram of Shandong circular high-speed railway

Fig 8. Revenue comparison under different price adjustment ranges

Fig 9. Ticket price fluctuation for different classes of trains under different OD classes

Fig 10. Revenue comparison under different heuristic algorithm parameters

6. It is “A to E” rather than “A to B” on line 194.

Response: Thanks for your valuable suggestion.

The incorrect text description has been fixed.

(Line 289-292)

In summary, there are 14 travel routes for the passengers from node A to E including another 4 two-time transfer routes. The quantity of service routes in such a simple service network is evidently a little much, and it will go up very sharply with the increase of the number of trains in the network.

7. There are 5 nodes in Fig. 3 and 4 four nodes in Fig. 4. Is there any correspondence between Fig. 3 and Fig. 4?

Response: Thanks for your kind question.

There are no correspondence between Fig. 3 and Fig. 4. They are different forms of train service network, aiming to explain the train service network construction based on timetable. We have modified the expression letters of passenger nodes in Fig. 3 from "A~D" to "S_1_~S_2_". At the same time, the text description is added to avoid causing ambiguity.

(Line 293-301)

In order to reduce the scale of train service network search, trains are converted to nodes and the main part of abstracted service network is comprised of trains nodes and transfer arcs. In addition, passengers enter or exit the network by passenger nodes and getting on/off arcs, as shown in Fig 3. Fig 2 and 3 are intended to illustrate the different forms of train service network, and there is no correspondence between them.

Fig 3. Abstracted train service network

8. The passenger time value is set as 50 yuan per person per hour in this study (line 238). How is it determined? What is the source of this value?

Response: Thanks for your kind question.

The passenger time value is calculated by Eq(5)

(5)

where *GDP* represents the regional economic development level, *P* represents the regional population, *LT* represents the regional per capita annual labor time, *LR* represents the regional average labor ratio.

Considering the case used the passenger flow data and timetable data in 2019, we calculated the passenger time value with 2019 data.

Table 1 is the initial calculation of passenger time value. Data source of *GDP* and *P* were "National Bureau of Statistics for 2019" and "Shandong Statistical Yearbook for 2019". It was reported that Chinese workers work 2,000 to 2,200 hours per year. Data source of *LT* and *LR* were estimated at 0.7 and 2000, respectively.

Table 1 Initial calculation of passenger time value

| Region | *GDP*(*trillion yuan*) | *P*(*million*) | *LT* | *LR*(*h*) | $\kappa_{w}$(*yuan/h*) |
| --- | --- | --- | --- | --- | --- |
| *Shandong* | 7.05 | 10070 | 0.7 | 2000 | 50.01 |
| *China* | 98.6515 | 141000 | 0.7 | 2000 | 49.98 |

As reminded by Reviewer#1, China's labor ratio should be lower than 0.7. We try to estimate the values of *LT* and *LR* from official data.

According to "Statistical Bulletin of the National Economic and Social Development of the People's Republic of China for 2019" and "Statistical Bulletin of the National Economic and Social Development of Shandong Province for 2019", population aged 15-64 is considered as the working population, and *LT* in Shandong province and China in 2019 were 0.66 and 0.64, respectively.

Due to the lack of accurate annual working hours, rough calculations are made based on scattered data. According to the National Bureau of Statistics, the average weekly working hour in June 2024 was 48.6 hours, up 2.9 hours from the same month in 2019. Deduce backwards from this, the average weekly working hour in 2019 was considered to be 45.7 hours, and the average annual working hour was calculated as 2193.6 hours, which was rounded up as 2190 hours.

After correcting *LT* and *LR*, Table 2 is obtained.

Table 2 Corrected calculation of passenger time value

| Region | *GDP*(*trillion yuan*) | *P*(*million*) | *LT* | *LR*(*h*) | $\kappa_{w}$(*yuan/h*) |
| --- | --- | --- | --- | --- | --- |
| Shandong province | 7.05 | 10070 | 0.66 | 2190 | 48.44 |
| China | 98.6515 | 141000 | 0.64 | 2190 | 49.92 |

In terms of the value of passenger time value, shandong province was lower than the national average, which seems to be counterintuitive.

In fact, in terms of per capita consumption expenditure for 2019, the national average and the Shandong average were 21,559 yuan and 20,427 yuan respectively, which supported the results in Table 2.

To avoid misunderstanding, expand the description. In terms of per capita disposable income of residents for 2019, the national average and the Shandong average were 30,733 yuan and 31,597 yuan respectively, which was in line with people's common sense. It showed that people in Shandong were more willing to save money than those in other regions.

Since the *Shandong circular high-speed railway* not only includes the passenger flow in Shandong province, in order to ensure the availability of data, we calculated the passenger time value of China and Shandong province respectively.

According to the "Compilation of National Railway Statistical Data in 2019", the passenger flow of Jinan Railway Bureau is shown in Table 3.

Table 3 Passenger flow of Jinan Railway Bureau in 2019

| Passenger flow type | Number (*million*) |
| --- | --- |
| Local | 9192 |
| Export | 6011 |
| Import | 6050 |
| Carrying | 9241 |

The local passengers and export passengers correspond to the passenger time value of Shandong province. The import passengers and carrying passengers correspond to the passenger time value of China.

According to the proportion of different types of passenger flow, the passenger time value was 49.18 yuan/h, which was rounded up to 50 yuan/h.

(Line 338-353)

Since the *Shandong circular high-speed railway* not only includes the passenger flow in Shandong province, in order to ensure the availability of data, the passenger time value of China and Shandong province were calculated respectively. Table 3 is the calculation of passenger time value.

Table 3. Calculation of passenger time value

| Region | *GDP*(*trillion yuan*) | *P*(*million*) | *LT* | *LR*(*h*) | $\kappa_{w}$(*yuan/h*) |
| --- | --- | --- | --- | --- | --- |
| Shandong province | 7.05 | 10070 | 0.66 | 2190 | 48.44 |
| China | 98.6515 | 141000 | 0.64 | 2190 | 49.92 |

Data source: National Bureau of Statistics for 2019, Shandong Statistical Yearbook for 2019, Statistical Bulletin of the National Economic and Social Development of the People's Republic of China for 2019, Statistical Bulletin of the National Economic and Social Development of Shandong Province for 2019.

Table 4 is the Passenger flow of Jinan Railway Bureau in 2019. The local passengers and export passengers correspond to the passenger time value of Shandong province. The import passengers and carrying passengers correspond to the passenger time value of China.

Table 4. Passenger flow of Jinan Railway Bureau in 2019

| Passenger flow type | Number (*million*) |
| --- | --- |
| Local | 9192 |
| Export | 6011 |
| Import | 6050 |
| Carrying | 9241 |

Data source: Compilation of National Railway Statistical Data in 2019.

According to the proportion of different types of passenger flow, the passenger time value was 49.18 yuan/h, which was rounded up to 50 yuan/h.

9. Equation 14 represents price not inverted constraint in a simple example. Please provide a general expression of the price not inverted constraint in a general situation.

Response: Thanks for your valuable comments.

We have made corrections in section 3(Key notations definition) and section 5.2(Decision variable and constraints), and have removed the constraint description of the original simple example.

(Line 246)

**Table 2. Notations definition table**

| **Notation** | **Definition** |
| --- | --- |
| *n* | The number of station intervals of OD *w* in route *k* |
| *l* | The OD corresponding to the adjacent station interval, $l\in[1,n],l\in Z^{+}$ |
| $p_{l,k,s}$ | The ticket price of OD *l* to seat *s* in route *k* |

(Line 414-418)

(2) Price not inverted constraint

Ticket prices should not be reversed, which means the price of a short-distance train ticket is lower than the price of a long-distance train ticket. If the total distance is equal, the combined value of two short-distance train ticket is not less than that of one long-distance train ticket.

$\sum_{l} p_{l,k,s}\geq p_{w,k,s},p_{l,k,s}\leq p_{w,k,s},l\in[1,n]$ (14)

10. The second objective function aims to maximize passenger travel benefit and solve the passenger assignment. Does Z2 consider the relationship between demand and the number of passengers? For example, the number of passengers should be smaller than the demand. How is the relationship between demand and assigned passenger flow reflected in this paper?

Response: Thanks for your kind question.

The question is very meaningful. Theoretically, when the ticket price changes, the passenger flow demand will change, and the elastic demand function should be used to describe the passenger flow demand.

In the case of this paper, we discussed this and finally chose to use the fixed real passenger flow demand for the following reasons:

1) From the perspective of the national network, *Shandong circular high-speed railway* is relatively independent in the overall high-speed railway network of China, and has limited influence on passenger flow demand of other lines. The reason is that Shandong Peninsula is coastal and geographically protruder.

In terms of lines, it only overlaps with *Beijing-Shanghai high-speed rail*way (*Jinan West* - *Qufu East*). In terms of nodes, only *Jinan* is regarded as a large node of passenger flow exchange with the trunk high-speed railway network.

From the perspective of line and train, our case study object is the circular high-speed trains of *Shandong circular high-speed railway*. The train alternative set (12 trains) is not large.

In the case of small train alternative set and small impact on the overall network, if the train timetable and ticket price are optimized at the same time, the change of multiple factors (such as ticket price, departure time, stop station, etc.) must consider the change of passenger demand. If only the ticket price is adjusted, the ticket price as a single factor has little influence on the total passenger flow demand.

In the case of large train alternative set and large impact on the overall network (such as *Beijing-Shanghai high-speed railway*), only the adjustment of ticket price must consider the change of passenger demand.

2) China's national conditions have determined that the railway has public welfare and publicness. China's high-speed railway not only needs to meet the travel demand of passengers, but also demand of social welfare. The policy of low fare rate were implemented for a long time, resulting in huge pressure on profitability of most lines.

Since 2020, China Railway has gradually implemented the dynamic pricing mechanism for high-speed. Railway related enterprises can implement dynamic pricing according to season, holidays, weekdays, weekends and seat levels, which has stimulated the enthusiasm of participating in market competition to a certain extent. However, the current dynamic pricing mechanism is still in the initial stage, and most of them are small-range floating strategies.

Considering the current national conditions, although we compared *LNS* (large neighborhood search) parameter from 10% to 100% in section 6.5, we do not set the *LNS* parameter to 100% in both section 6.3 and section 6.4. In most cases, not all trains carry out dynamic pricing, which further reduces the impact of fare as a single factor on the total passenger flow demand.

11. Equation 18 represents the transfer times constraint? It is suggested to provide the calculation formula of the number of transfer times.

Response: Thanks for your kind question.

Because passengers are discrete, it is difficult to quantify the maximum number of transfer times accepted by formula.

Our survey questionnaire for *Shandong circular high-speed railway* in 2019 was aimed at designing tourism products and did not involve the maximum number of transfer times accepted.

Considering that the *Beijing-Shanghai High-Speed Railway* overlaps with the *Shandong circular high-speed railway* (*Jinan West* - *Qufu East*), there is a small gap in passenger travel characteristics in the eastern region, taking the questionnaire survey of *Beijing-Shanghai high-speed railway* in February 2024 as an example, the maximum number of transfer times acceptable to passengers under different travel miles is calculated, as shown in Table 4.

The reason for choosing the 2024 questionnaire is that the COVID-19 has had a great impact on people's travel, and the data for 2020-2023 may be uncertain.

Table 4 Passenger transfer times intentions

| Travel miles | The maximum number of  transfer times accepted | Passengers | Ratio | |
| --- | --- | --- | --- | --- |
| ＜500km | 0 | 308 | 40.7% | **82.3%** |
|  | 1 | 315 | 41.6% |  |
|  | ＞1 | 134 | 17.7% | 17.7% |
| 500-1000km | 0 | 259 | 34.2% | **72.9%** |
|  | 1 | 293 | 38.7% |  |
|  | ＞1 | 205 | 27.1% | 27.1% |
| ＞1000km | 0 | 193 | 25.5% | **67.5%** |
|  | 1 | 318 | 42.0% |  |
|  | ＞1 | 246 | 32.5% | 32.5% |

As can be seen from Table 4, the proportion of passengers who can accept the maximum number of transfer times less than 2 is as high as 82.3% within (0,500] km, 72.9% within (500,1000] km, and 67.5% above 1000km.

In terms of line length, *Jiaoji HSR* is 365.5km, *Jiqing HSR* is 307.9 km, *Rilan HSR*(*Rizhao West - Qufu East* section) is 235 km, *Jinghu HSR* (*Jinan west - Qufu east* section) is 129 km, *Qingyan HSR* in Shandong province is about 186.6 km, the total length of *Shandong circular high-speed railway* is less than 1300 km, of which *Jiaoji HSR* and *Jiqing HSR* are parallel.

In terms of passenger flow, not all passengers will take a complete circle. The passenger flow between Qingdao and Jinan accounts for relatively large proportion, and the distance of two key transportation nodes is about 300-360km. This can be seen in the following table.

Table 5 Departure and arrival passenger flow of stations

| Departure Station | Departure PF | Arrival Station | Arrival PF |
| --- | --- | --- | --- |
| *Qingdao North* | 71999 | *Qingdao North* | 67339 |
| *Jinan West* | 62214 | *Jinan West* | 63733 |
| *Jinan* | 60146 | *Jinan* | 58715 |
| *Qingdao* | 28264 | *Qingdao* | 28537 |
| *Qufu East* | 27669 | *Qufu East* | 26629 |
| *Weifang* | 25121 | *Weifang* | 25918 |
| *Jinan East* | 24212 | *Jinan East* | 22920 |
| *Zibo* | 20326 | *Zibo* | 20552 |
| *Taian* | 19683 | *Rizhao West* | 17549 |
| *Rizhao West* | 17331 | *Taian* | 17392 |
| *Linyi North* | 15408 | *Linyi North* | 15841 |
| *Weifang North* | 8403 | *Weifang North* | 8011 |
| *Zibo North* | 7724 | *Zibo North* | 7948 |
| *Qingzhoushi* | 6970 | *Qingzhoushi* | 6873 |
| *Qingdao West* | 6611 | *Qingdao West* | 6599 |
| *Zhangqiu* | 5592 | *Zhangqiu* | 5452 |
| *Gaomi* | 5112 | *Gaomi* | 4818 |
| *Daminghu* | 4412 | *Daminghu* | 3614 |
| *Mengshan* | 2528 | *Mengshan* | 2929 |
| *Zouping* | 2431 | *Zouping* | 2502 |
| *Feixian North* | 2116 | *Feixian North* | 2172 |
| *Jiaozhou North* | 1739 | *Jiaozhou North* | 2150 |
| *Junan North* | 1734 | *Junan North* | 1973 |
| *Linzi North* | 1642 | *Linzi North* | 1447 |
| *Changle* | 1311 | *Gaomi North* | 1370 |
| *Zhangqiu North* | 1264 | *Changle* | 1333 |
| *Dongjiakou* | 855 | *Zhangqiu North* | 1158 |
| *Gaomi North* | 601 | *Dongjiakou* | 895 |
| *Lijiazhai* | 469 | *Lijiazhai* | 535 |
| *Sishui South* | 332 | *Sishui South* | 460 |

The Departure and arrival passenger flow of stations are counted and arranged in order from largest to smallest, as shown in Table 5. No matter the departure passenger flow or the arrival passenger flow, the top four stations belong to Jinan node and Qingdao node. Because *Jinan East* is far away from the urban area, its departure and arrival passenger flow both rank seventh. For the same reason, although *Qingdao West* belongs to Qingdao administratively, it is very far away from the urban area, so the departure and arrival passenger flow both are small.

In summary, we consider it is reasonable to set the maximum number of passenger transfer times to 1.

Due to the length and structure of article, we have not included all of the above analysis in the revised manuscript, but have added a text description to explain why we set the maximum number of acceptable passenger transfer times to 1.

(Line 436-442)

(6) Transfer times constraint

When the capacity of direct service routes is insufficient, passengers will tend to choose transfer service routes. However, too many transfer times will against the travel experience of passengers. According to the investigation of the maximum passenger acceptable transfer times in relevant areas and the passenger flow analysis of *Shandong circular high-speed railway*, the maximum acceptable transfer times is set to be no more than 1.

$h_{w,k,s}\leq1$ (18)

12. In section 5.3, it is suggested to remove the step 2 in the solution steps.

Response: Thanks for your valuable comments.

In combination with No.12 and No.13 comments, we have supplemented the detailed steps of the global algorithm structure. Specific changes are as follows:

(1) The original step 2 was deleted.

(2) Considering that the *LNS* algorithm in this paper is deeply related to the ticket price adjustment strategy, the initial writing is not easy for people to understand until section 6.2 of the case. We completely separate the ticket price adjustment strategy from the *LNS* algorithm.

(3) The ticket price adjustment strategy is further divided into two parts: train classification (New step 2) and subdivision ticket price adjustment strategy (New step 3).

(4) The operation description of the *LNS* algorithm is improved in step 4, and the details in subsequent steps are corrected.

(Line 452-489)

Step 1: Input initial data, including line price rate ***R_0_***, dynamic pricing range **[*LowerRate*, *UpperRate*]**, total iterations times ***T***, convergence value **△*c*** , OD passenger flow data, train timetable data.

Step 2: Establish the classification of trains based on multiple factors(such as number or proportion of different level stations, etc). Single or multiple factors are considered, depending on the availability of relevant data.

Step 3: Establish the subdivision ticket price adjustment strategy based on train class.

- Step 3.1: Set different price gaps. The positions of different gaps are different. The practical significance of the narrow gap is greater, and the research significance of the wide gap is greater.
- Step 3.2: The ticket price adjust ranges of different class trains under different gaps are determined. The higher the train class, the higher the ticket price rate.

Step 4: Carry out the large neighborhood search(*LNS*) algorithm.

- Step 4.1: Select ***P*** percent of the trains randomly for ***Destroy*** and ***Repair*** operations. The value of ***P*** can be deterministic or random.
- Step 4.2: ***Destroy*** means the selected trains(***P*** percent quantity and reserved integer) are removed from the train alternative set. The ticket price rate of the selected trains will be adjusted randomly within the range by the above strategy. The ticket price rate of other trains in the alternative set remains unchanged.
- Step 4.3: ***Repair*** means the ticket price rate of the selected trains has been adjusted. And then they are inserted into the train alternative set.

Step 5: Carry out the path size logit(*PSL*) assignment algorithm based on capacity constraint-passenger flow increment(*CCPFI*).

- Step 5.1: Search for direct routes and transfer routes for different ODs based on train timetable data.
- Step 5.2: For different routes of different ODs, calculate route utility and path size logit choice probability based on train price rate (i.e. the route selection ratio).
- Step 5.3: Considering OD classes, OD passenger flow demand and train capacity, simulate passenger travel choice behavior. If the train capacity is sufficient, the number of passengers on board is the product of route selection ratio and OD passenger flow demand. Otherwise, the number of passengers on board is the train surplus capacity.
- Step 5.4: Output enterprise income ***x*** and passenger flow assignment results.

Step 6: Evaluate the output result against the current global optimal solution ***x^b^***. Determine whether the iteration times ***T*** is reached. If true, go Step 9. Otherwise, go Step 7.

Step 7: If **| *x* - *x^b^* |*≤*△*c***, go Step 9. Otherwise, go Step 8.

Step 8: If ***x≥x^b^***, update the current global optimal solution ***x^b^ =x*** .Then, go Step 4.

Step 9: End the loop and output ***max*{ *x*, *x^b^* }**.

13. How is the LNS algorithm designed (Step 3)? The clear algorithm steps and solving ideas should be given.

Response: Thanks for your kind question.

In combination with comment 12 and comment 13, we have supplemented the detailed steps of the global algorithm structure. Specific changes are as follows:

(1) The original step 2 was deleted.

(2) Considering that the *LNS* algorithm in this paper is deeply related to the ticket price adjustment strategy, the initial writing is not easy for people to understand until section 6.2 of the case, we completely separate the ticket price adjustment strategy from the *LNS* algorithm.

(3) The ticket price adjustment strategy is further divided into two parts: train classification (New step 2) and subdivision ticket price adjustment strategy (New step 3).

(4) The operation description of the *LNS* algorithm is improved in step 4, and the details in subsequent steps are corrected.

(Line 452-489)

Step 1: Input initial data, including line price rate ***R_0_***, dynamic pricing range **[*LowerRate*, *UpperRate*]**, total iterations times ***T***, convergence value **△*c*** , OD passenger flow data, train timetable data.

Step 2: Establish the classification of trains based on multiple factors(such as number or proportion of different level stations, etc). Single or multiple factors are considered, depending on the availability of relevant data.

Step 3: Establish the subdivision ticket price adjustment strategy based on train class.

- Step 3.1: Set different price gaps. The positions of different gaps are different. The practical significance of the narrow gap is greater, and the research significance of the wide gap is greater.
- Step 3.2: The ticket price adjust ranges of different class trains under different gaps are determined. The higher the train class, the higher the ticket price rate.

Step 4: Carry out the large neighborhood search(*LNS*) algorithm.

- Step 4.1: Select ***P*** percent of the trains randomly for ***Destroy*** and ***Repair*** operations. The value of ***P*** can be deterministic or random.
- Step 4.2: ***Destroy*** means the selected trains(***P*** percent quantity and reserved integer) are removed from the train alternative set. The ticket price rate of the selected trains will be adjusted randomly within the range by the above strategy. The ticket price rate of other trains in the alternative set remains unchanged.
- Step 4.3: ***Repair*** means the ticket price rate of the selected trains has been adjusted. And then they are inserted into the train alternative set.

Step 5: Carry out the path size logit(*PSL*) assignment algorithm based on capacity constraint-passenger flow increment(*CCPFI*).

- Step 5.1: Search for direct routes and transfer routes for different ODs based on train timetable data.
- Step 5.2: For different routes of different ODs, calculate route utility and path size logit choice probability based on train price rate (i.e. the route selection ratio).
- Step 5.3: Considering OD classes, OD passenger flow demand and train capacity, simulate passenger travel choice behavior. If the train capacity is sufficient, the number of passengers on board is the product of route selection ratio and OD passenger flow demand. Otherwise, the number of passengers on board is the train surplus capacity.
- Step 5.4: Output enterprise income ***x*** and passenger flow assignment results.

Step 6: Evaluate the output result against the current global optimal solution ***x^b^***. Determine whether the iteration times ***T*** is reached. If true, go Step 9. Otherwise, go Step 7.

Step 7: If **| *x* - *x^b^* |*≤*△*c***, go Step 9. Otherwise, go Step 8.

Step 8: If ***x≥x^b^***, update the current global optimal solution ***x^b^ =x*** .Then, go Step 4.

Step 9: End the loop and output ***max*{ *x*, *x^b^* }**.

14. In step 4, the number of passengers on board appears to be allocated rather than optimized by the second objective function.

Response: Thanks for your valuable comments.

The *PSL*(path size logit) model is an improvement of the *MNL* model and follows random utility theory.

In the consumer choice behavior, each choice has a corresponding utility, which includes two parts: the observable part and the unobservable part (usually called random error term).

By assuming that the random error term follows some distribution (such as the Gumbel distribution), the above models express the choice probability as a function of the observable part of the utility, and solve the function to get the probability of each option being chosen.

Therefore, the *PSL* model and the *MNL* model do not directly solve an explicit objective function (such as maximizing total utility or minimizing cost), but indirectly reflect the utility maximization behavior by calculating the choice probability.

The following is a extended description of the path size logit passenger flow assignment algorithm strategy for imitating passenger travel behavior.

In the travel choice behavior of passengers, at the individual level, passengers tend to choose the route (i.e. the train or trains) that is most effective for them. At the overall level, we simulate passenger choice behavior through OD classification, out-of-order assignment and other passenger flow assignment strategies in the algorithm to ensure the accuracy of train-flow as far as possible.

The role of passenger flow assignment strategy is explained, which can try to avoid the following problems:

1) Long-distance train capacity is occupied by short-distance ODs.

2) Single OD occupies too much capacity of the same train.

3) Part of passenger flow cannot be assigned to the ideal train because the order is later.

The passenger flow assignment strategy is a part of path size logit passenger flow assignment algorithm. Considering that the main innovation of this paper is dynamic pricing optimization based on train classification, and if it is to be fully explained, the algorithm will take up too much space, so it is not described in the manuscript.

15. “high-speed” of the legend in Fig. 7 should be replaced by “HSR”.

Response: Thanks for your valuable comments.

The legend description in Fig.7 has been modified.

(Line 553-554)

Fig 7. The schematic diagram of Shandong circular high-speed railway

16. Train classification is achieved according to the station levels rather than train attributes (or service level) such as speed, departure time, price and so on. What are the advantages of the classification method in this paper compared to the classification method according to train service level. Why do authors choose this classification method?

Response: Thanks for your kind question.

The purpose of train classification is to determine different ticket price adjustment ranges according to the clustering results.

There are two difficulties of the train classification based on service attributes.

Firstly, the number of train service attributes is large, including the departure time, arrival time, train travel speed, the number of train stops, ticket prices, comfort and so on. And there is no fixed criterion to judge the quality of some attributes. Take departure time as an example, different passengers have different preferences to it, it is difficult to explain which time of departure train is the best.

Secondly, the contribution rate (or parameters) of various service attributes is difficult to quantify. It is difficult to score and rank the competitiveness of each train in a formula.

To solve the first difficulty, the train classification based on station levels transforms the train service attributes into station attributes. The station attributes mainly include the number of trains served, the number of passengers served and the administrative level of the city.

In terms of quantity, station attributes contains fewer elements than the train service attributes. In terms of structure, the above three factors have clear levels. It is easy to divide and manage the station levels.Take the number of passengers served as an example, the station level with a high number of passengers served must be higher than the station level with a small number of passengers served.

To solve the second difficulty, the train classification based on station levels transforms the quantification problem of train service attribute parameters into the proportion or correlation matrix problem of different level stations, which greatly reduces the difficulty of train classification.

In terms of classification results, the higher the proportion of high-level stations, it means that the number of train stops is less, the travel speed is faster, the arrival and departure time is usually better, thus reflecting the train service attributes.

In theory, both the train classification based on service attributes and the train classification based on station levels can be classified by clustering software (such as SPSS), but the classification standard is not clearly defined in this way, just like a black box. We hope to get a train classification method that not only has a clear methodology, but also can reduce the difficulty of practical operation. Therefore, we chose the train classification based on station levels.

17. In the numerical studies, the revenue should be compared between two cases. Case 1 represents the train classification. Case 2 represents no train classification with the uniform price upper and lower bounds. This comparison could help reveal the meaning of this train classification method.

Response: Thanks for your valuable comments.

1) For the problem of different upper and lower bounds, we have made modifications. The specific value range can be found in the response to Reviewer#2's No.18 comment.

2) For the case revenue comparison of whether the train is classified or not, we added the comparison case in the case revenue analysis (section 6.3). However, this is not a comparative case of whether the train is classified or not, but a comparative case of whether the ticket price adjustment strategy is adopted.

The explanation of why this is done follows.

Firstly, explain the reason for the classification of trains.

China has gradually loosened the control of high-speed railway ticket price in recent years. However, the current dynamic pricing mechanism is still in the initial stage, mostly small ranges of floating strategy based on subjective experience. There are some problems, such as lack of scientific theoretical support, failure to fully segment the market and products, and slow process of overall marketization. In addition, the methods of dynamic pricing proposed in few relevant literatures are difficult to apply.

Based on the present situation, we would like to propose a ticket price adjustment strategy with high practical and theoretical support. Therefore, train classification is combined with dynamic pricing. The train classification is related to the number of stations and station class, which ensures the high practicality of the method. Front-line railway staff can focus on station class, ticket price adjustment range, etc., instead of studying the complex model and algorithm of dynamic pricing.

Secondly, explain the choice of comparative cases.

Take the OD "*Linyi North* - *Jinan*" as an example, the train *G5525* and *G5535* belong to ClassⅠand ClassⅡrespectively, and the specific timetables are as follows.

Table 6 The timetable of *G5525* (ClassⅠ)

| TrainNo | Station | ArrTime | DepTime | Miles |
| --- | --- | --- | --- | --- |
| **G5525** | ***Linyi North*** | **8:00** | **8:00** | **0** |
| G5525 | *Feixian North* | 8:13 | 8:16 | 43 |
| G5525 | *Mengshan* | 8:27 | 8:30 | 72 |
| G5525 | *Qufu East* | 8:51 | 8:53 | 134 |
| G5525 | *Taian* | 9:13 | 9:15 | 204 |
| **G5525** | ***Jinan*** | **9:38** | **9:40** | **266** |
| G5525 | *Zibo* | 10:22 | 10:24 | 376 |
| G5525 | *Weifang* | 10:59 | 11:01 | 476 |
| G5525 | *Qingdao North* | 11:59 | 12:18 | 644 |
| G5525 | *Qingdao West* | 12:54 | 12:56 | 718 |
| G5525 | *Dongjiakou* | 13:13 | 13:15 | 753 |
| G5525 | *Rizhao West* | 13:37 | 13:40 | 797 |
| G5525 | *Lijiazhai* | 13:53 | 13:56 | 827 |
| G5525 | *Junan North* | 14:07 | 14:10 | 857 |

Table 7 The timetable of *G5535* (ClassⅡ)

| TrainNo | Station | ArrTime | DepTime | Miles |
| --- | --- | --- | --- | --- |
| **G5535** | ***Linyi North*** | **13:56** | **13:56** | **0** |
| G5535 | *Feixian North* | 14:09 | 14:11 | 43 |
| G5535 | *Mengshan* | 14:22 | 14:24 | 72 |
| G5535 | *Sishui South* | 14:37 | 14:39 | 100 |
| G5535 | *Qufu East* | 14:52 | 14:55 | 134 |
| **G5535** | ***Jinan*** | **15:43** | **15:46** | **266** |
| G5535 | *Zhangqiu* | 16:09 | 16:11 | 315 |
| G5535 | *Weifang* | 17:12 | 17:15 | 476 |
| G5535 | *Jiaozhou North* | 17:52 | 17:54 | 596 |
| G5535 | *Qingdao North* | 19:29 | 19:49 | 674 |
| G5535 | *Lijiazhai* | 21:11 | 21:13 | 857 |

According to the timetables, the service time of the OD "*Linyi North* - *Jinan*" is 1 hour 38 minutes(*G5525*) and 1 hour 47 minutes(*G5535*) respectively. If the train class is not divided, the ticket price adjustment range of *G5525* and *G5535* is the same, and there may be a situation that the ticket price of the slower train is more expensive, which is unreasonable.The point is that this is not reflected in the revenue, so we do not compare the revenue of trains classified versus unclassified.

However, it is reasonable to add comparative cases. After revising the ticket price adjustment strategy based on subsequent comments, we have added the case of non-variable ticket price in the total revenue analysis (section 6.3).

Due to the change of specific ticket price strategy, all cases have been recalculated and analyzed, and the specific case values have changed.

(Line 617-629)

The national conditions determine that high-speed railway enterprises must assume political tasks and social missions while pursuing the maximization of profits with the goal of marketization. The fixed ticket price mechanism formed under the government guidance for a long time involves many factors in railway transportation management, resulting in the current dynamic pricing mechanism is relatively strict control. The actual maximum price fluctuation gap in the eastern developed region is about 20%.

Based on this, we set price gap to a maximum of 40% and a minimum of 20%, with the same upper and lower bounds. The overall range based on train classification is half of the price gap. According to the station decision matrix, the subdivide range is half or all of the overall range. The specific ticket price adjustment strategies of *Shandong circular high-speed railwa*y are shown in Table 8.

Table 8. Specific ticket price adjustment strategy of *shandong circular high-speed railway*

| Price Gap | Train Class | Overall Range | Decision Matrix | Subdivide Range |
| --- | --- | --- | --- | --- |
| 40% | Class Ⅰ | 0 ~ 20% | [1,1,0] | 10% ~ 20% |
|  |  |  | [1,0,0] | 0 ~ 20% |
|  | Class Ⅱ | -10% ~ 10% | [1,0,1] | 0 ~ 10% |
|  |  |  | [0,1,0] | -10% ~ 10% |
|  | Class Ⅲ | -20% ~ 0 | [0,1,1] | -10% ~ 0 |
|  |  |  | [0,0,1] | -20% ~ 0 |
| 30% | Class Ⅰ | 0 ~ 15% | [1,1,0] | 7.5% ~ 15% |
|  |  |  | [1,0,0] | 0 ~ 15% |
|  | Class Ⅱ | -7.5% ~ 7.5% | [1,0,1] | 0 ~ 7.5% |
|  |  |  | [0,1,0] | -7.5% ~ 7.5% |
|  | Class Ⅲ | -15% ~ 0 | [0,1,1] | -7.5% ~ 0 |
|  |  |  | [0,0,1] | -15% ~ 0 |
| 20% | Class Ⅰ | 0 ~ 10% | [1,1,0] | 5% ~ 10% |
|  |  |  | [1,0,0] | 0 ~ 10% |
|  | Class Ⅱ | -5% ~ 5% | [1,0,1] | 0 ~ 5% |
|  |  |  | [0,1,0] | -5% ~ 5% |
|  | Class Ⅲ | -10% ~ 0 | [0,1,1] | -5% ~ 0 |
|  |  |  | [0,0,1] | -10% ~ 0 |

(Line 644-655)

In order to verify the effectiveness of the model and algorithm under different price adjustment ranges(*Series1*[-10%,10%], *Series2*[-15%,15%], *Series3*[-20%,20%]), the ***Destroy*** and ***Repair*** operations ratio in the large neighborhood search is set to 50%, and the income curves under different price adjustment ranges are drawn respectively, as shown in Fig 8.

In terms of the revenue curve, with the increase of the ticket price adjustment range, the absolute income and the fluctuating range increases. The reason is that the upper and lower bounds of the ticket price floating range are the same, and they are gradually increasing.

In terms of average income, compared with *original price*, the average income increase rate of *Series1, Series2* and *Series3* is 3.63%, 5.9% and 7.92% respectively.

**Fig 8. Revenue comparison under different price adjustment ranges**

18. How is the subdivide range in Table 5 determined?

Response: Thanks for your kind question.

China's national conditions determine that China's dynamic pricing mechanism is complicated and challenging.

First of all, the national conditions determine that high-speed railway enterprises must assume political tasks and social missions while pursuing the maximization of profits with the goal of marketization, which leads to some restrictions on the line types, train types, and floating ranges applicable to dynamic pricing.

Secondly, the theoretical and practical basis needs to be further accumulated. The fixed ticket price mechanism formed under the government guidance for a long time involves many factors in railway transportation management, resulting in the current dynamic pricing mechanism is relatively strict control. The actual maximum price fluctuation gap in the eastern developed region is about 20%.

Based on the above national conditions and Reviewer#2's No.17 comment and No.20 comment, we have revised the original specific ticket price adjustment strategy.

1) The price gap is divided into three categories, which are 20% (the actual maximum gap of the current policy), 30%, and 40% (the theoretical maximum gap of strong competitiveness and suitable for market-oriented high-speed railway).

2) The overall range based on train classification is half of the price gap.

3) The subdivide range based on the the station decision matrix is half or all of the overall range.

We have supplemented the value of the specific ticket price adjustment strategy.

(Line 617-629)

The national conditions determine that high-speed railway enterprises must assume political tasks and social missions while pursuing the maximization of profits with the goal of marketization. The fixed ticket price mechanism formed under the government guidance for a long time involves many factors in railway transportation management, resulting in the current dynamic pricing mechanism is relatively strict control. The actual maximum price fluctuation gap in the eastern developed region is about 20%.

Based on this, we set price gap to a maximum of 40% and a minimum of 20%, with the same upper and lower bounds. The overall range based on train classification is half of the price gap. According to the station decision matrix, the subdivide range is half or all of the overall range. The specific ticket price adjustment strategies of *Shandong circular high-speed railwa*y are shown in Table 8.

Table 8. Specific ticket price adjustment strategy of *shandong circular high-speed railway*

| Price Gap | Train Class | Overall Range | Decision Matrix | Subdivide Range |
| --- | --- | --- | --- | --- |
| 40% | Class Ⅰ | 0 ~ 20% | [1,1,0] | 10% ~ 20% |
|  |  |  | [1,0,0] | 0 ~ 20% |
|  | Class Ⅱ | -10% ~ 10% | [1,0,1] | 0 ~ 10% |
|  |  |  | [0,1,0] | -10% ~ 10% |
|  | Class Ⅲ | -20% ~ 0 | [0,1,1] | -10% ~ 0 |
|  |  |  | [0,0,1] | -20% ~ 0 |
| 30% | Class Ⅰ | 0 ~ 15% | [1,1,0] | 7.5% ~ 15% |
|  |  |  | [1,0,0] | 0 ~ 15% |
|  | Class Ⅱ | -7.5% ~ 7.5% | [1,0,1] | 0 ~ 7.5% |
|  |  |  | [0,1,0] | -7.5% ~ 7.5% |
|  | Class Ⅲ | -15% ~ 0 | [0,1,1] | -7.5% ~ 0 |
|  |  |  | [0,0,1] | -15% ~ 0 |
| 20% | Class Ⅰ | 0 ~ 10% | [1,1,0] | 5% ~ 10% |
|  |  |  | [1,0,0] | 0 ~ 10% |
|  | Class Ⅱ | -5% ~ 5% | [1,0,1] | 0 ~ 5% |
|  |  |  | [0,1,0] | -5% ~ 5% |
|  | Class Ⅲ | -10% ~ 0 | [0,1,1] | -5% ~ 0 |
|  |  |  | [0,0,1] | -10% ~ 0 |

19. It is suggested to present the optimal results, such as the ticket price for each seat class between each OD served by each train.

Response: Thanks for your valuable comments.

In section 6.4 of the case study, we summarized the average iterated ticket prices of for key ODs of different trains with different classes.

(Line 657-665)

The basis of enterprise ticketing and passenger ticket purchase is the ticket price with ODs as the unit. Select “*Linyi North*-*Qingdao North*”, “*Qingdao West*-*Qufu East*”, “*Dongjiakou-Mengshan*” as the key ODs of different levels based on Table 6.

When the price adjustment range is set to [-20%,20%], the ***Destroy*** and ***Repair*** operations ratio in the large neighborhood search is set to 40%, remove unreasonable detour trains and calculate the price fluctuation range of different trains under various ODs. The average iterated ticket prices for key ODs of different classes are shown in Table 9. The box plots are drawn, as shown in Fig 9.

Table 9 Average iterated ticket prices for key ODs of different classes

| OD Level | DepSta | ArrSta | TrainNo | Ticket Price(*yuan*) |
| --- | --- | --- | --- | --- |
| Large OD | *Linyi North* | *Qingdao North* | *G5526* | 103.3 |
|  |  |  | *G5536* | 99.8 |
|  |  |  | *G5567* | 110.9 |
| Medium OD | *Qingdao West* | *Qufu East* | *G5555* | 125.1 |
|  |  |  | *G5565* | 130.6 |
|  |  |  | *G6955* | 122.5 |
|  |  |  | *G6975* | 122.8 |
| Small OD | *Dongjiakou* | *Mengshan* | *G5555* | 86.5 |
|  |  |  | *G6955* | 84.2 |
|  |  |  | *G6975* | 84.9 |

20. In section 6.3, the price adjustment ranges have different upper bounds and uniform lower bounds. It is suggested to add different lower bounds to reflect the optimization rules more comprehensively.

Response: Thanks for your valuable comments.

The different upper and lower bounds of the ticket price adjustment range have been fixed. The specific value range can be found in the response to Reviewer#2's No.18 comment.

Due to the change of specific ticket price strategy, all cases need to be recalculated and analyzed. We have compiled the revised case study (section 6.3 to section 6.5) in the response.

Because there are so many changes, we put the case analysis (section 6.3 to section 6.5) as a whole below.

(Line 634-739)

**6.3 Ticket price comparison analysis**

In order to verify the effectiveness of the model and algorithm under different price adjustment ranges(Series1[-10%,10%], Series2[-15%,15%], Series3[-20%,20%]), the Destroy and Repair operations ratio in the large neighborhood search is set to 50%, and the income curves under different price adjustment ranges are drawn respectively, as shown in Fig 8.

In terms of the revenue curve, with the increase of the ticket price adjustment range, the absolute income and the fluctuating range increases. The reason is that the upper and lower bounds of the ticket price floating range are the same, and they are gradually increasing.

In terms of average income, compared with original price, the average income increase rate of Series1, Series2 and Series3 is 3.63%, 5.9% and 7.92% respectively.

**Fig 8. Revenue comparison under different price adjustment ranges**

**6.4 Ticket price comparison analysis**

The basis of enterprise ticketing and passenger ticket purchase is the ticket price with ODs as the unit. Select “*Linyi North*-*Qingdao North*”, “*Qingdao West*-*Qufu East*”, “*Dongjiakou-Mengshan*” as the key ODs of different levels based on Table 6.

When the price adjustment range is set to [-20%,20%], the ***Destroy*** and ***Repair*** operations ratio in the large neighborhood search is set to 40%, remove unreasonable detour trains and calculate the price fluctuation range of different trains under various ODs. The average iterated ticket prices for key ODs of different classes are shown in Table 9. The box plots are drawn, as shown in Fig 9.

Table 9 Average iterated ticket prices for key ODs of different classes

| OD Level | DepSta | ArrSta | TrainNo | Ticket Price(*yuan*) |
| --- | --- | --- | --- | --- |
| Large OD | *Linyi North* | *Qingdao North* | *G5526* | 103.3 |
|  |  |  | *G5536* | 99.8 |
|  |  |  | *G5567* | 110.9 |
| Medium OD | *Qingdao West* | *Qufu East* | *G5555* | 125.1 |
|  |  |  | *G5565* | 130.6 |
|  |  |  | *G6955* | 122.5 |
|  |  |  | *G6975* | 122.8 |
| Small OD | *Dongjiakou* | *Mengshan* | *G5555* | 86.5 |
|  |  |  | *G6955* | 84.2 |
|  |  |  | *G6975* | 84.9 |

In the box plots, the data above the box indicates that the size is sorted in the first 25%, the data below the box indicates that the size is sorted in the last 25%, the data box indicates that the size is sorted between 25%-75%. The upper boundary of the box represents the upper quartile and the lower boundary represents the lower quartile. The whiskers are lines extending from the box and cover 99.3% of the data volume. The upper and lower boundaries of the whiskers represent the maximum and minimum values of the data, respectively. MEAN value is the literal meaning. Outliers represent data which exceeds the whiskers.

Fig 9. Ticket price fluctuation for different classes of trains under different OD classes

The ticket price fluctuation of different trains(ClassⅠ*G5526* and Class Ⅲ *G5536*) under large OD (*Linyi North*-*Qingdao North*) is calculated, as shown on the left side of Fig 9. In terms of ticket price dynamic range, the box range of *G5536* is obviously narrower than that of G*5526*, indicating that the ticket price of *G5536* is relatively stable and according with the basic situation that the ticket price fluctuation range of ClassⅠis larger than that of Class Ⅲ. In terms of MEAN value, the average ticket price of *G5536* is lower than that of *G5526*. In terms of whiskers, *G5526* has only upper whisker and *G5536* has only lower whisker. The reason is that ClassⅠonly consider ticket price rate increase while Class Ⅲ only consider ticket price rate decrease Table 8.

The ticket price fluctuation of different trains(ClassⅠ *G5565* and ClassⅡ*G6975*) under medium OD (*Qingdao West*-*Qufu East*) is calculated, as shown in the middle of Fig 9. In terms of ticket price dynamic range, the box range of *G5565* is wider than that of *G6975*, matching the basic situation that the ticket price fluctuation range of ClassⅠis larger than that of mudiun train. In terms of MEAN value, the average ticket price of *G5565* is higher than that of *G6975*. The reason why the MEAN value of *G6975* close to the box is that some outliers are small, which lowers the average ticket price. Generaly speaking, as long as the MEAN value is within two whiskers, the result can be reasonably analyzed. In terms of whiskers, G5556 lacks lower whisker, which is consistent with the ticket price adjustment range in Table 8.

The ticket price fluctuation of different trains(ClassⅡ *G6975* and Class Ⅲ *G6955*) under small OD (*Dongjiakou-Mengshan*) is calculated, as shown on the right of Fig 9. In terms of price dynamic range, the box range of *G6975* and *G6955* is similar. In terms of MEAN value, the average ticket price of *G6975* is higher than that of *G6955*. In terms of whiskers, G6955 lacks upper whisker, aligning with the ticket price adjustment range in Table 8.

**6.5 Algorithm core parameter analysis**

The core parameter of large neighborhood search algorithm is the ratio of ***Destory*** and ***Repair*** operations for standby sets. Generally speaking, the higher the ratio, the more trains change price rate in each cycle.

In large-scale neighborhood search, if the parameter setting is too small, only a small part of the solutions are destroyed and repaired, then the heuristic search is difficult to search the whole neighborhood. If the parameter setting is too large, most of the solutions are broken, then the heuristic algorithm almost degenerates into repeated re-optimization.

In the scenario where the price adjustment range is set as [-20%,20%], destory and repair parameters are set at 10% intervals. The average income value and calculation solving time curve are shown in Fig 10.

Fig 10. Revenue comparison under different heuristic algorithm parameters

In terms of average revenue, as the parameters ratio increases, the average revenue increases steadily. In terms of calculation time, as the parameters ratio increases, the curve shows an upward trend, and the value increases exponentially.

Among them, when the parameter increases from 50% to 60%, the calculation time increases slowly and the average revenue decreases slightly, indicating that 60% is an important reference node. As data scales up, railway staff should maintain a balance between total revenue and calculation time.

21. It is suggested that the future research direction should be more detailed and specific.

Response: Thanks for your valuable comments.

We have located the current research to the station and train dimension, and look forward to the future research in different dimensions, and give examples.

(Line 751-761)

This paper studies the ticket price adjustment strategy of high-speed railway from the dimensions of station and train. Future research can be conducted from the following two aspects:

The first is to continue in-depth optimization in the dimensions of station and train. According to different scenarios, different station classification and train classification methods can be proposed. For the same class of trains, the factors(such as historical passenger flow, historical turnover, etc) can be combined to further refine the ticket price adjustment strategy.

The second is to further expand the train dimension to the pre-sale period dimension and OD dimension. In the dimension of pre-sale period, the ticket price adjustment strategy can be studied on a daily basis or a multi-hour basis. In the OD dimension, different OD ticket price rates of the same train may fluctuate differently.
