# Supplementary material for: Dynamic pricing optimization for high-speed railway based on passenger flow assignment
Source: PLoS One. 2024 Dec 17;19(12):e0314713. doi: 10.1371/journal.pone.0314713 (PMC11651575; doi:10.1371/journal.pone.0314713)
Supplement: Attachment — Submitted filename: Response to Reviewers.docx [file pone.0314713.s004.docx]

Dear editor and reviewers,

On behalf of my co-authors, I extend our heartfelt gratitude for the opportunity to revise our submission. We are deeply appreciative of the insightful and constructive feedback provided on our manuscript, titled "Dynamic Pricing Optimization for High-Speed Railway Based on Passenger Flow Assignment" (PONE-D-24-26027R1).

Your comments have been invaluable in enhancing the quality of our work and have offered significant guidance for our ongoing research endeavors. We have meticulously reviewed each point and have made the necessary revisions, which we believe address the concerns raised. We hope that our amendments will be found satisfactory.

Note: In the response letter, the explanatory text is in blue, the revised content in the manuscript is in green. ‘L’ shows the index of lines in the ‘Revised Manuscript with Track Changes’.

Best wishes,

CAO Jiren

Reviewer #3：

Your study tackles an important issue in the transportation sector by proposing a dynamic pricing model that considers passenger flow assignment. The integration of differentiated train products and dynamic pricing strategies is particularly interesting and has the potential to contribute significantly to the field.
However, I have several concerns that need to be addressed before the manuscript can be considered for publication. Below are detailed technical questions and comments that highlight areas of ambiguity and require clarification.

1. Model Justification: Can you elaborate on the rationale behind choosing the specific algorithms for passenger flow assignment? What criteria were used to evaluate their effectiveness?

Response: Thank you for your meaningful question.

The uniqueness of the passenger assignment algorithm proposed in this paper lied in its classification and adjustment of OD level. In addition, during the train-flow matching process, it set the maximum passenger flow assignment number per time, rather than allowing all passengers of the current OD to board at once. And the ODs were arranged out of order before each passenger flow assignment.

We have optimized the passenger flow assignment algorithm chart and placed it below. Next, we will explain the existing problems with traditional passenger assignment algorithms and the reasons for adopting the aforementioned strategy.

Firstly, we divide the OD levels according to station classes, one of the reasons for which is that the high level ODs have more passenger flow than low level ODs. Besides, the overlap degree of long-distance ODs and high-level ODs is high. If long-distance ODs are not assigned firstly, the train capacity may be occupied by short-distance ODs, and it is difficult for passengers of long-distance ODs to board. This is consistent with the priority sale of long-distance tickets in reality.

Secondly, we adjust OD levels based on supply and demand. If a certain OD can only take a certain train to meet the travel demand, the level of this OD should be elevated to avoid the problem that the passengers of this OD cannot board.

Thirdly, we set the maximum passenger flow assignment number per time according to OD levels. Due to the limited train capacity, the passenger flow assignment in turn of different ODs may be unreasonable, such as a train serving only single OD or a few ODs which have large passenger flow. The maximum passenger flow assignment number per time can avoid the problem that single OD occupies too much capacity of the same train.

Finally, we sort all ODs with passenger flow greater than 0 in random order. The random assignment can prevent the situation where part of passenger flow cannot be assigned due to being at the end of the queue.

Fig 6. Path Size Logit Assignment based on Capacity Constraint-Passenger Flow Increment Algorithm

On the whole, the optimization purpose of the passenger flow assignment algorithm is to simulate the actual choice behavior of passengers, and to avoid the situations that obviously will not occur in reality through the algorithm strategy.

In terms of passenger flow assignment evaluation index, the average train occupancy rate is taken as the standard. When the price adjustment range is set to [-20%, 20%], the ratio of Destroy and Repair operations in the large neighborhood search is set to 40%, the average train occupancy rate increased significantly from 34.08% to 44.72%.

The reason for the overall low occupancy rate is that the case data comes from the trial operation in October 2019. At that time, the *Shandong circular high-speed railway* was not fully operational, resulting in relatively low overall passenger flow.

Considering the overall structure of the paper, the evaluation indexes of passenger flow assignment are not emphasized in the manuscript.

2. Dynamic Pricing Mechanism: How does the dynamic pricing mechanism adapt to real-time changes in passenger demand? Are there specific thresholds or triggers that initiate price adjustments?

Response: Thank you for your meaningful question.

For the dynamic pricing mechanism of high-speed railway to adapt to the real-time changes in passenger demand, it must necessarily involve price fluctuations during the pre-sale period. Outside the pre-sale period, the railway ticketing system is unable to capture the real-time demand of passengers.

The research scenario of this paper is the dynamic pricing mechanism outside the pre-sale period, using actual historical passenger flow data as the demand for passenger flow, without considering the real-time changes in passenger demand.

The deep reason for the above positioning is related to the current national conditions and market-oriented reforms of China's high-speed railway. China's unique national conditions make the high-speed railway dynamic pricing mechanism more complex and challenging than other countries and regions.

First of all, China's national conditions determine that high-speed railway enterprises must undertake social welfare while pursuing the maximum profit with the goal of marketization. As a result, the applicable scope of the dynamic pricing strategy is limited, such as the line types, the train types, and the dynamic pricing range.

Secondly, the theory and practice of high-speed railway dynamic pricing need to be further accumulated. There are many factors in railway transportation management, so the current mechanism is mostly small ranges of dynamic pricing strategy based on subjective experience, which lacks scientific theoretical support.

Under such a background, the overall process of China's railway marketization is slow, and the ticket prices of lines have not formed scientific and effective adjustment methods.

From the perspective of research, the dynamic pricing outside the pre-sale period is the basis of the dynamic pricing during the pre-sale period. The latter requires additional consideration of constraints (such as daily seat allocation, passenger flow transfer between different dates, and the heterogeneity of passengers between different periods, etc.) which will suddenly increase in difficulty.

From the perspective of reform, the initial stage of reform needs to be steady, first aiming at the dynamic pricing outside the pre-sale period, laying a good research foundation, and then further studying the price fluctuation during the pre-sale period.

To sum up, this paper is positioned in the scenario of dynamic pricing outside the pre-sale period. Future studies could take into account the real-time demand of passengers and add trigger conditions to initiate price adjustments.

(Line 62-65)

From the perspective of scientific research, the dynamic pricing outside the pre-sale period is the basis of the dynamic pricing during the pre-sale period. From the perspective of market-oriented reform, the initial stage of reform needs to be steady. Therefore, the scenario is set to the dynamic pricing outside the pre-sale period.

3. Data Sources: What specific operational data were used in your case study? How was the data validated to ensure its reliability?

Response: Thank you for your meaningful question.

In the case study, we utilized train timetable data and passenger flow data, both of which are real data from the Jinan Railway Bureau on October 25, 2019.

The attributes of the train timetable data include train number, station stop order, station name, departure time, arrival time, distance traveled, and train capacity.

The attributes of the passenger flow data include train number, departure station, arrival station, and passenger flow volume.

The aforementioned data have been submitted to the PLOS ONE system, named respectively as "S1 Table. Train Timetable Data" and "S2 Table. Passenger Flow Data". The reliability of the data can be verified through these two documents. Table 1 and Table 2 are examples of train timetable data and passenger flow data respectively.

Table 1 Sample table of train timetable data

| Trainno | No | Sta | DepTime | ArrTime | Miles | Capcity |
| --- | --- | --- | --- | --- | --- | --- |
| *G5525* | 1 | *Linyi North* | 8:00 | 8:00 | 0 | 1112 |
| *G5525* | 2 | *Feixian North* | 8:13 | 8:16 | 43 | 1112 |
| *G5525* | 3 | *Mengshan* | 8:27 | 8:30 | 72 | 1112 |
| *G5525* | 4 | *Qufu East* | 8:51 | 8:53 | 134 | 1112 |
| *G5525* | 5 | *Taian* | 9:13 | 9:15 | 204 | 1112 |
| *G5525* | 6 | *Jinan* | 9:38 | 9:40 | 266 | 1112 |
| *G5525* | 7 | *Zibo* | 10:22 | 10:24 | 376 | 1112 |
| *G5525* | 8 | *Weifang* | 10:59 | 11:01 | 476 | 1112 |
| *G5525* | 9 | *Qingdao North* | 11:59 | 12:18 | 644 | 1112 |
| *G5525* | 10 | *Qingdao West* | 12:54 | 12:56 | 718 | 1112 |
| *G5525* | 11 | *Dongjiakou* | 13:13 | 13:15 | 753 | 1112 |
| *G5525* | 12 | *Rizhao West* | 13:37 | 13:40 | 797 | 1112 |
| *G5525* | 13 | *Lijiazhai* | 13:53 | 13:56 | 827 | 1112 |
| *G5525* | 14 | *Junan North* | 14:07 | 14:10 | 857 | 1112 |

Table 2 Sample table of passenger flow data

| Osta | Dsta | PF |
| --- | --- | --- |
| *Mengshan* | *Linyi North* | 1387 |
| *Jinan* | *Linyi North* | 408 |
| *Jinan* | *Linyi North* | 324 |
| *Linyi North* | *Jinan* | 315 |
| *Jinan* | *Qingdao North* | 276 |
| *Linyi North* | *Jinan* | 275 |
| *Jinan* | *Linyi North* | 267 |
| *Jinan West* | *Linyi North* | 238 |
| *Qingdao North* | *Linyi North* | 222 |
| *Jinan* | *Qingdao North* | 183 |
| *Linyi North* | *Qingdao North* | 176 |
| *Taian* | *Qingdao North* | 172 |

4. Algorithm Complexity: The manuscript mentions the use of a large-scale neighborhood search algorithm. Can you provide more details on its implementation and computational complexity?

Response: Thank you for your meaningful question.

Large Neighborhood Search (*LNS*) algorithm, as a heuristic search algorithm, explores the solution space through destroy and repair operations to find better solutions.

The purpose of the destroy operation is to disrupt a part of the current solution to generate new candidate solutions. In the case study, for the train alternative set, based on the *LNS* core parameter ratio (Section 6.5), a portion of trains are randomly selected and removed from the alternative set.

The repair operation follows the destroy operation, with the aim of reconstructing the disrupted solution to make it feasible again. In the case study, for trains removed from the alternative set, based on the ticket price adjustment strategy (Section 6.2), their ticket price rates are adjusted, and the trains with the changed ticket price rates are reinserted into the train alternative set.

In terms of algorithm complexity, the *LNS* algorithm proposed in this paper is nested with the *PSL* passenger flow assignment algorithm, and its complexity is polynomial.

The overall algorithm is embedded in three sets of loops. The first loop is for repeated calculations, not involving the core algorithm, with the purpose of taking the average total revenue as the result. The second loop is related to the OD number (denoted by ***n***), and the third loop is related to the train number (denoted by ***m***). Therefore, the overall algorithm complexity is polynomial, related to the OD number ***n*** and the train number ***m***.

5. Passenger Flow Assignment: How do you ensure that the passenger flow assignment accurately reflects real-world scenarios? Are there any assumptions made that could impact the results?

9. Passenger Behavior Insights: How does your model account for passenger behavior and preferences? Are there any behavioral assumptions that could influence the pricing outcomes?

Response: Thank you for your meaningful question.

Both Comment #5 and Comment #9 involve to passenger choice behavior and relevant assumptions. We will answer them in detail together.

In the study of passenger flow assignment, the model is strongly related to the algorithm.

Regarding "how passenger flow assignment reflects real-world scenarios" and "how the model account for passenger behavior and preferences", we will provide explanations from both the model and algorithm perspectives.

With respect to the model, the core model of passenger flow assignment is *PSL* (Path Size Logit) model. The *PSL* model is an improvement of the *MNL* (Multinomial Logit) model and follows random utility theory.

According to the random utility theory, in the consumer choice behavior, each choice has a corresponding utility, which includes two parts: the observable part and the unobservable part (usually called random error term).

By assuming that the random error term follows some distribution, the above models express the choice probability as a function of the observable part of the utility, and solve the function to get the probability of each option being chosen.

*MNL* model and its derived models have been verified and widely used in China's transportation research, including Fu, Jin and Lin(2010), Zhang, Jian and Zhu(2015), Sun and Zheng(2016), Hu, Yao and Liu(2017), Wang, Liu and Liu(2020), Li, Yang and Ding(2022), etc.

[1].	Fu, W., W. Jin and F. Lin, Calculation of Time Value for Urban Public Travel with MNL Model. Journal of Transportation Systems Engineering and Information Technology, 2010. 10(02): p. 148-152.

[2].	Zhang, R., W. Jian and L. Zhu, Reliability Analysis of Railway Container Transport Time. Journal of the China Railway Society, 2015.

[3].	Sun, Q. and X. Zheng, The Transportation Corridor Mode Choice Model Based On the Interaction Mechanism of Transportation Service Level, Operational Efficiency and Resource Consumption. Journal of Transportation Systems Engineering and Information Technology, 2016. 16(01): p. 26-31.

[4].	Hu, J., M. Yao, Y. Liu, L. Tang and J. Chen, Research On the Travel Choice Behaviors of Transportation Corridor Based On Sem-MNL Model. Journal of Railway Engineering Society, 2017.

[5].	Wang, J., J. Liu and Y. Liu, Research On the Development Model of Urban and Rural Road Passenger Transport Integration Based On Rank Analysis and Multinomial Logit Model. Journal of Railway Science and Engineering, 2020. 17(12): p. 3224-3230.

[6].	Li, J., G. Yang, Y. Ding and J. Liu, Impacts of a New Rail Transit Line On Travel Mode Choice. Journal of Transportation Systems Engineering and Information Technology, 2022. 22(05): p. 135-140+153.

With respect to the algorithm, in the response to Comment #1, we explained the particularities of the passenger flow assignment algorithm in this paper (including OD level, the maximum number of passenger flow assignment per time, random assignment, etc.), with the aim of simulating the actual ticketing situation in China and passenger choice behavior.

Facing the background of numerous passenger ODs and tight train capacity, especially in the eastern coastal region, China railway prioritizes the sale of tickets for long-distance and major node connections during ticketing. This is the reason why we differentiate between OD level.

In the actual selection process of passengers, there won't be a situation where a single train serves only a few ODs or even a single OD. Taking into account the competitive game, the vast majority of passengers are actually able to buy the tickets they desire. This is why we set a maximum number of passenger flow assignment per time and implement random assignment.

Regarding "Are there any assumptions made that could impact the results or influence the pricing outcomes", we will analyze the assumptions (Section 5) that may impact the results and explain why we chose to make such assumptions.

The model proposed in this paper assumes that the travel demand of passengers is independent and does not affect each other. This assumption could have a certain impact on the results, such as in group travel, where passengers can influence each other's choice behavior.

The main reason for making such an assumption lies in the ticket selling method of Chinese railways. Railway 12306 website (including mobile client) is the only official online ticketing platform of China Railway. When passengers buy tickets, they need to correspond with their ID cards one by one. Even group travel tickets are displayed in the 12306 system as separate ticket purchases. In addition, group travel accounts for a relatively low proportion of overall passenger demand.

Therefore, we believed that the aforementioned assumption could have a certain impact on the results, but it was not significant, and we retained the assumption.

(Line 298-306)

Railway 12306 website (including mobile client) is the only official online ticketing platform of China Railway. When passengers buy tickets, they need to correspond with their ID cards one by one. Even group travel tickets are displayed in the 12306 system as separate ticket purchases. Additionally, Chinese high-speed railway only allow a very limited number of trains to carry a few extra passengers during busy holidays.

Based on the above national conditions, the following assumptions are made:

The model in this paper makes the following assumptions:

(1) The model does not consider overbooking.

(2) The travel needs of passengers are independent and do not affect each other.

(Line 418-421)

According to the random utility theory of passenger choice behavior, By assuming that the random error follows identical and independent(IID) Gumbel distribution, path size logit assignment algorithm based on capacity constraint-passenger flow increments calculated using Eq(19) where $U_{w,k,s}$, $U_{w,m,s}$ are given in Eq(8).

6. Sensitivity Analysis: Have you conducted any sensitivity analysis to understand how changes in key parameters affect the outcomes of your model?

Response: Thank you for your meaningful question.

In the *LNS* algorithm, the destroy and repair parameters are key parameters that together define the neighborhood structure of *LNS*.

The destroy method increases the diversity of the search space by breaking the current solution, while the repair method attempts to find a better solution within the broken solution.

In this paper, destroy means the selected trains (*P* percent quantity and reserved integer) are removed from the train alternative set. The ticket price rate of the selected trains will be adjusted randomly within the range by the ticket price adjustment strategy. The ticket price rate of other trains in the alternative set remains unchanged. Repair means the ticket price rate of the selected trains has been adjusted. And then they are inserted into the train alternative set.

In Section 6.5, sensitivity analysis was performed for destroy and repair parameters. We have revised the wording in Section 6.5 to ensure that the message conveyed is accurate.

(Line 574-604)

**6.5 Algorithm core parameter sensitivity analysis**

In the *LNS* algorithm, the destroy and repair parameters are key parameters that together define the neighborhood structure of *LNS*. The destroy method increases the diversity of the search space by breaking the current solution, while the repair method attempts to find a better solution within the broken solution. Generally speaking, the higher the ratio of destroy and repair operations, the more trains change price rate in each cycle.

In large-scale neighborhood search, if the parameter setting is too small, only a small part of the solutions are destroyed and repaired, then the heuristic search is difficult to search the whole neighborhood. If the parameter setting is too large, most of the solutions are broken, then the heuristic algorithm almost degenerates into repeated re-optimization.

In the scenario where the price adjustment range is set as [-20%,20%], destroy and repair parameters are set at 10% intervals. The average income value and calculation solving time curve are shown in Fig 10.

**Fig 10. Revenue comparison under different heuristic algorithm parameters**

In the context of average revenue, a steady increase is observed as the parameter ratio escalates. With respect to calculation time, an exponential growth is evident as the parameter ratio is augmented.

Particularly, upon incrementing the parameter ratio from 50% to 60%, there is a modest acceleration in calculation time, concomitant with a marginal decline in average revenue. This observation underscores the significance of the 60% threshold as a critical reference point.

As the scale of data intensifies, it becomes imperative for railway staff to achieve a delicate equilibrium between optimizing total revenue and managing calculation time.

7. Comparison with Existing Models: How does your proposed model compare with existing dynamic pricing models in terms of performance and applicability? Are there any benchmarks used for comparison?

Response: Thank you for your meaningful question.

One of the innovations of the proposed model presented in this paper lies in the integration of dynamic pricing with passenger flow assignment, with dynamic pricing serving as the primary optimization objective.

Existing research on dynamic pricing of high-speed railway is mostly related to revenue management and seat allocation. Existing research on passenger flow allocation is mostly related to passenger choice behavior. There are few literatures considering dynamic pricing and passenger flow assignment at the same time. And these few literatures focus on analyzing passenger choice behavior under different ticket prices, which means that ticket prices are optimization conditions rather than optimization purposes.

Another innovation lies in the incorporation of several practical railway operational requirements (such as price not inverted constraint, transfer times constraint, etc.) without oversimplifying the model through excessive assumptions. Consequently, the model proposed in this paper is characterized by its broad applicability, ease of operation, and practical alignment.

In the context of actual railway operations, the comparative benchmarks for different models should include two components: total revenue and operational cost. The former can be represented in numerical form, while the latter is difficult and challenging to quantify and compare.

8. Implementation Challenges: What are the potential challenges in implementing your proposed pricing strategy in a real-world setting? How do you suggest overcoming these challenges?

Response: Thank you for your meaningful question.

According to the response 2, China's high-speed railway had long implemented a low ticket price rate policy. The variable ticket price policy, which is designed to respond dynamically to market conditions, will primarily encounter resistance from passengers who are accustomed to lower ticket price rates.

To navigate this challenge, it is imperative that the dynamic pricing adjustments are balanced, incorporating both increases and decreases, rather than focusing solely on upward adjustments.

The balance in ticket price adjustments is crucial to ensure that the high-speed railway dynamic pricing mechanism can achieve economic benefits while also taking into account social equity. Besides, it ensures that the ticket price policy is sustainable and equitable.

10. Economic Implications: Can you discuss the broader economic implications of your pricing strategy on the high-speed railway market and its stakeholders?

Response: Thank you for your meaningful question.

Taking the data of 2019 as an example, the high-speed railway passenger volume reached 2.29 billion. On the demand side, there were a total of 81960 passenger flow ODs. Among them, the revenue of 7800 passenger flow ODs was 278.9 billion yuan. The number of passenger flow ODs accounted for 9.5%, contributing to 87% of the total revenue. On the supply side, the passenger volume of China Railway Shanghai Group Co., Ltd which located in the eastern region accounted for 26% of the total passenger volume. The passenger volume of Beijing-Shanghai high-speed railway contributed to more than 10% of the total revenue.

In terms of economic development, the top 7800 passenger flow ODs were mostly distributed on busy lines or intercity lines. The time-sensitive business passenger flow had a significant proportion. If the fares of these passenger flow are raised by 1% on average, the revenue can be increased by 2.79 billion yuan, which has huge economic benefits.

As one of the initial attempts of China's railway market-oriented reform, this paper only studies the *Shandong circular high-speed railway*. If the effectiveness of the ticket price adjustment strategy is proved in the actual operation and the expected benefits are obtained, it can be extended to small-scale networks and eventually cover the whole network. Then, the ticket price adjustment strategy can optimize passenger travel options and facilitate the efficient allocation of transportation resources, but also can promote the sustainable development of the high-speed railway industry.

11. Limitations of the Study: What are the limitations of your study, and how might they affect the generalizability of your findings?

Response: Thank you for your meaningful question.

In terms of limitations, due to the availability of data, the ticket price adjustment strategy proposed in this paper only studied second-class seats and did not include business-class and first-class seats.

Compared to second-class passengers, those in business-class and first-class seats are more sensitive to time and less sensitive to price. Take a single seat for example, the revenue increase from dynamic pricing for business-class and first-class seats can be higher than that for second-class seats.

However, in actual operations, the number of business-class and first-class seats is far less than that of second-class seats. Without considering special trains, the number of business-class seats in a train will not exceed 10. In an 8-carriages train, there is usually only 1 first-class carriage. In a 16-carriages train, there may be 2 first-class carriages. The majority of the carriages are designated as second-class.

Therefore, in terms of economies of scale, the expected revenue from dynamic pricing for business-class and first-class seats is likely to be less than that for second-class seats.

This does not mean that there is no need to study the dynamic pricing for business-class and first-class seats. Instead, scientific research should initially focus on the dynamic pricing for second-class seats, which cover the majority of passengers. Based on this foundation and in response to market feedback, a detailed study of dynamic pricing for business-class and first-class seats can be conducted.

It should be noted that the research approach for different classes of seating is the same, with the emphasis on distinguishing the heterogeneity of the target passengers. The acceptable range of dynamic pricing varies among different passengers, which means that the specific ticket price adjustment strategies for different types of seating must be tailored accordingly.

(Line 457-464)

Due to the availability of data, only second-class seats are considered in case. It should be noted that the research approach for different classes of seating is the same, with the emphasis on distinguishing the heterogeneity of the target passengers. The acceptable range of dynamic pricing varies among different passengers. Therefore, the second-class seat scenario case can still verify the applicability of the model and algorithms which can still be applied to the scenarios considering different seats.

12. Future Research Directions: What future research directions do you envision based on the findings of your study? Are there specific areas that require further exploration?

Response: Thank you for your meaningful question.

In response to your suggestions and questions, we have expanded the future research directions in Section 7 (Conclusion).

(Line 615-629)

This paper studies the ticket price adjustment strategy of high-speed railway from the dimensions of station and train. Future research can be conducted from the following aspects:

(1) Refine ticket price adjustment strategies at station level and train level. According to different scenarios, different station classification and train classification methods can be proposed. For the same seat class, the factors (such as historical passenger flow, historical turnover, etc.) can be combined. For the different seat classes, different ticket price adjustment ranges can be set.

(2) Expand ticket price adjustment strategies at the pre-sale period and OD level. In the dimension of pre-sale period, the ticket price adjustment strategy can be studied on a daily basis or a multi-hour basis and adapted to real-time changes in passenger demand. In the OD level, different OD ticket price rates of the same train may fluctuate differently.
